# Analgosedation during flexible fiberoptic bronchoscopy: comparing the clinical effectiveness and safety of remifentanil versus midazolam/propofol

**DOI:** 10.1186/s12890-019-1004-6

**Published:** 2019-12-09

**Authors:** Hyun Lee, Yeong Hun Choe, Seungyong Park

**Affiliations:** 1 Division of Pulmonary Medicine and Allergy, Department of Internal Medicine, Hanyang University Hospital, Seoul, Republic of Korea; 2 Department of Internal Medicine, Chonbuk National University Hospital-Chonbuk National University Medical School, Jeonju, Republic of Korea; 3 Research Institute of Clinical Medicine of Chonbuk National University-Biomedical Research Institute of Chonbuk National University Hospital, Jeonju, Republic of Korea

**Keywords:** Remifentanil, Midazolam, Propofol, Bronchoscopy, Analgosedation

## Abstract

**Background:**

There are limited data regarding the efficacy and safety of remifentanil sedation for diagnostic bronchoscopy. The aim of this study was to evaluate the clinical efficacy and safety of remifentanil by comparing it with those of conventional drugs, midazolam and propofol.

**Methods:**

A retrospective study of 186 patients who underwent diagnostic bronchoscopy at Chonbuk National University Hospital was performed. Patients were classified into the remifentanil group and midazolam/propofol group according to the drugs used during bronchoscopy.

**Results:**

Of the 186 patients, 111 patients received remifentanil and 75 received midazolam/propofol during the bronchoscopy. The proportion of patients who required bronchoscopy for endobronchial inspection alone was significantly higher in the midazolam/propofol group than in the remifentanil group (93.3% vs. 73.0%; *p* <  0.001). In contrast, the proportion of patients who required more invasive procedures, such as bronchoscopic biopsy, bronchoalveolar lavage, or transbronchial lung biopsy, was significantly higher in the remifentanil group than in the midazolam/propofol group (27.0% vs. 6.7%; *p* <  0.001). The recovery time was significantly shorter in the remifentanil group than in the midazolam/propofol group (mean 6.4 min vs. 11.6 min, *p* <  0.001). There were no significant differences between the groups with regard to safety events including desaturation, hypotension, and arrhythmia.

**Conclusions:**

Despite the higher proportion of patients who underwent more invasive procedures in the remifentanil group than in the midazolam/propofol group, there was no significant difference in safety events between the groups. Those in the remifentanil group also demonstrated a faster recovery time than those in the midazolam/propofol group.

## Background

Flexible bronchoscopy remains the gold standard for numerous diagnostic and therapeutic interventions and has become an integral part of pulmonary medicine [1]. Given its widespread use, it is important to select appropriate sedation to relieve the anxiety and pain of patients undergoing bronchoscopy [2–4]. The type of sedation may also be helpful for the bronchoscopist to facilitate the procedure [4].

Although the current guidelines recommend the combined use of an opioid and midazolam for bronchoscopy sedation [3], physicians often use a single drug, or a combination of benzodiazepines, propofol, and opioids in this setting [5, 6]. In addition, with the development of new drugs, remifentanil and dexmedetomidine have been introduced as options for analgosedation for flexible bronchoscopy in adults [7–10]. Accordingly, the combination of new drugs and conventional drugs has enabled a wide variety of combinations of drugs to be used for analgosedation for flexible bronchoscopy. Several studies have demonstrated the safety and efficacy of the addition of remifentanil or dexmedetomidine (to conventional drugs) in adult patients who underwent flexible bronchoscopy [7–10]. However, the best-standardized practice for the use of sedation during bronchoscopy has yet to be determined.

Remifentanil is an ultra short-acting new opioid analgesic drug that is commonly used to relieve pain during the surgery and as adjunctive to an anesthetic [11]. The ultrafast offset and short half-life also make it an ideal drug for analgosedation in intensive care unit (ICU) patients [12]. Considering that remifentanil has excellent analgosedation effects with an advantage of the short half-life, remifentanil could become an alternative to conventional treatments (including the combination of a benzodiazepine and propofol) for sedation in bronchoscopy. However, few studies have compared the effectiveness and safety profile of remifentanil versus a combination of midazolam and propofol. Therefore, the aim of this study was to compare the effectiveness and safety of analgosedation using remifentanil versus the combination of midazolam and propofol during flexible bronchoscopy.

## Methods

### Study design and population

This retrospective observational study was conducted at a tertiary university-affiliated hospital from May 2015 to September 2017. It was approved by the local Institutional Review Board (IRB no. CUH 2019–06-028). The patients were stratified into the remifentanil group and midazolam/propofol group according to the drugs used during flexible bronchoscopy.

### Flexible bronchoscopy

Flexible fiberoptic bronchoscopy was performed with standard fiberscopes by two professional pulmonologists. A flexible bronchoscope (BF-F260, BF-6C260, 1 T260, BF-Q290, 1TQ290, Olympus, Tokyo, Japan) was inserted via the nasal approach with subjects in the supine position.

All consecutive patients who were spontaneously breathing were eligible. All patients had fasted for at least 8 hours prior to the start of the procedure and had an intravenous catheter. Topical anesthesia was performed using 4% lidocaine spray in the oral cavity. While the vocal cords and carina were visualized, 4 ml of 2% lidocaine was delivered through the bronchoscope channel to suppress cough. Additional topical anesthesia was applied to the major bronchi as needed (at the bronchoscopists’ discretion) for a maximum total lidocaine dose of 7 mg/kg.

### Sedation

#### Midazolam/propofol group

Pretreatment with 1–2 mg midazolam (Midazolam®, Bukwang, Seoul, Republic of Korea) was used to relieve significant preprocedural anxiety. Patients were treated with an additional 1 mg of midazolam if necessary. Next, propofol (Anepol®, Hana Pharmaceutical Co., Seoul, Republic of Korea) was administered according to our hospital’s protocol.

**Table 1:** Baseline characteristics

	Remifentanil group (*n* = 111)	Midazolam / propofol group (*n* = 75)	*p*
Age, years	59.0 (51.0–70.5)	58.0 (42.0–71.5)	0.158
Sex, male	51 (45.9)	29 (38.7)	0.405
Body mass index, kg/m^2^	22.0 (20.0–24.0)	22.0 (20.0–24.5)	0.630
Comorbidities			
Hypertension	33 (29.7)	17 (22.7)	0.370
Diabetes mellitus	12 (10.8)	4 (5.3)	0.298
Asthma	1 (0.9)	2 (2.7)	0.730
Chronic obstructive pulmonary disease	2 (1.8)	2 (2.7)	1.000
Chronic heart disease	6 (5.4)	4 (5.3)	1.000
Pulmonary function			
FVC, L	2.9 (2.4–3.5)	3.1 (2.6–3.7)	0.129
FVC, % predicted	94.0 (85.0–102.0)	99.0 (88.5–107.5)	0.076
FEV_1_, L	2.3 (1.8–2.7)	2.4 (1.9–2.8)	0.538
FEV_1_, % predicted	99.0 (90.0–111.0)	102.5 (91.0–111.0)	0.691
FEV_1_/FVC	78.0 (73.0–82.5)	76.0 (71.0–82.5)	0.726

Data are present as number (%) or median (interquartile range)

*FVC* Forced expiratory volume, *FEV*_*1*_ Forced expiratory volume in 1 s

#### Remifentanil group

Patients received no premedication in the remifentanil group. Remifentanil (Ultiva®, GlaxoSmithKline, Seoul, Republic of Korea) was initially administered with a bolus dose of the study drug (0.5–1 mcg/kg), followed by a 0.02–0.04mcg/kg/min continuous infusion at the discretion of the bronchoscopist. A bolus dose of the study drug in proportional volumes was administered slowly (over a period of 60 s) to minimize known adverse effects, such as respiratory depression, hypotension, and bradycardia. The fiberscope was inserted into the patient 3–5 min after the remifentanil initial onset time. The remifentanil infusion was immediately stopped if bradypnea/apnea or hemodynamic collapse occurred.

### Outcome variables

The primary outcomes included the incidences of oxygen desaturation and oxygen saturation trend between the two groups. Oxygen desaturation was defined as an oxygen saturation < 90% for more than 10s. In the event of oxygen desaturation, oxygen delivery was increased from 4 to 10 L/min. Additional assistance (including reservoir bag-mask, high flow nasal cannula, and intubation followed by bag valve mask ventilation) was performed in a stepwise manner as needed. Secondary outcomes included hemodynamic variables, the incidence of hemodynamic adverse events, and recovery time.

Vital signs were continuously monitored before, during, and after the procedure by the bronchoscopist and nurses. Bradycardia (defined by a heart rate < 60 beats/min or a decrease of > 15 beats/min from baseline) was treated with intravenous atropine 0.5 mg. Hypotension (defined by a systolic blood pressure < 90 mmHg or a decrease of > 20 mmHg from baseline) was treated with intravenous epinephrine 5 mg. Bradypnea (defined by a respiratory rate < 12/min or apnea >10s), and other adverse reactions were noted throughout the procedure.

After the bronchoscopy, the patient was moved to the recovery room. In the recovery room, the recovery of the patient was assessed using the modified Aldrete score [13] every 3–5 min by the attending nurses. Recovery time was defined as the time taken after the end of bronchoscopy until the recovery, which was defined ≥9 for the modified Aldrete score [13]. Recovery time was classified as fast and delayed according to the median value of recovery time.

### Statistical analysis

Continuous variables are presented as mean (standard deviation [SD]) or median (interquartile range, [IQR]). Categorical variables are presented as counts (%). Continuous variables were compared using the Mann-Whitney U test or T-test for continuous variables. Categorical variables were compared using the chi-square test or Fischer’s exact test, as appropriate. In order to evaluate the factors associated with fast recovery time, we performed univariable and multivariable logistic regression analyses. The following factors were included in the multivariable model: age, sex, body mass index, chronic obstructive pulmonary disease or asthma, chronic heart disease, bronchoscopy indication, procedure, and type of sedative drugs. Two-sided *p* values < 0.05 were considered significant. All statistical analyses were performed using R (ver. 3.2.3; R Foundation for Statistical Computing, Vienna, Austria) and STATA version 15 (StataCorp LP, College Station, TX, USA).

## Results

### Patients

The baseline characteristics are summarized in Table 1. There were no significant differences between the two groups with regard to age, sex, body mass index, comorbidities such as hypertension, diabetes mellitus, asthma, chronic obstructive pulmonary disease, and chronic heart disease, or pulmonary function.

### Indication for bronchoscopy and use of sedative drugs

Infection, lung cancer, and hemoptysis were common indications of bronchoscopy in the remifentanil group in order of frequency. In comparison, infection, hemoptysis, and lung cancer were common indications of bronchoscopy in the midazolam/propofol group (Table 2). Patients in the remifentanil group were more likely to undergo invasive procedures during the bronchoscopy (beyond inspection alone) than were those in the midazolam/propofol group. The median procedure time was 9 min (IQR, 8–10 min) in the remifentanil group vs. 11 min (IQR, 10–13 min) in the midazolam/propofol group (*p* <  0.001). The remifentanil group received a median of 0.2 μg/kg/min (IQR, 0.18–0.33 μg/kg/min) of remifentanil. The midazolam/propofol group received a median of 2 mg of midazolam (IQR, 0–4 mg) and 50.2 µg/kg/min (39.0–64.3 μg/kg/min) of propofol.

**Table 2:** Indication for bronchoscopy and the use of sedative drugs

	Remifentanil group (*n* = 111)	Midazolam / propofol group (*n* = 75)	*p*
Indication for bronchoscopy			0.137
Infection	45 (40.5)	40 (53.3)	
Lung cancer	19 (17.1)	6 (8.0)	
Hemoptysis	13 (11.7)	12 (16.0)	
Interstitial lung disease	2 (1.8)	0 (0)	
Others	32 (28.8)	17 (22.7)	
Procedure			0.005
Endobronchial inspection only	81 (73.0)	70 (93.3)	
Endobronchial inspection with bronchoscopic biopsy	20 (18.0)	1 (1.3)	
Endobronchial inspection with BAL and TBLB	2 (1.8)	0 (0.0)	
Endobronchial inspection with BAL	8 (7.2)	4 (5.3)	
Procedure duration, min	9 (8–10)	11 (10–13)	< 0.001
Sedative drugs			
Midazolam dose, mg	–	2 (0–4)	
Propofol dose rate, ug/kg/min	–	50.2 (39.0–64.3)	
Remifentanil dose rate, ug/kg/min	0.2 (0.2–0.3)		

Data are present as number (%) or median (interquartile range)

*BAL* Bronchoalveolar lavage, *TBLB* Transbronchial lung biopsy

### Comparing the adverse events and recovery time between groups

There were no significant differences in oxygen desaturation, lowest peripheral capillary oxygen saturation, heart rate, antidote use, or sedation discontinuation between the remifentanil group and midazolam/propofol group. However, hypertension, defined by a blood pressure > 150/90 mmHg was significantly more common in the remifentanil group than it was in the propofol/midazolam group (52.3% vs. 30.7%; *p* = 0.008). In contrast, the recovery time was significantly lower in the remifentanil group than in the midazolam/propofol group (mean ± SD, 6.4 ± 3.0 min vs. 11.6 ± 4.0 min, *p* <  0.001) (Table 3).

**Table 3:** Comparing adverse events and recovery time across groups

	Remifentanil group (*n* = 111)	Midazolam / propofol group (*n* = 75)	*p*
Desaturation	9 (8.1)	6 (8.0)	1.000
Lowest SpO_2_	73.2 ± 12.3	81.8 ± 6.0	0.174
Heart rate			0.294
Tachycardia	18 (16.2)	17 (22.7)	
Bradycardia	5 (4.5)	1 (1.3)	
Blood pressure			0.008
Highest BP > 150/90 mmHg	58 (52.3)	23 (30.7)	
Lowest BP < 90/60 mmHg	0 (0)	1 (1.3)	
Recovery time, min	6.4 ± 3.0	11.6 ± 4.0	< 0.001
Antidote use	2 (1.8)	1 (1.3)	1.0
Sedation medication discontinuation	7 (6.3)	5 (6.7)	1.0

Data are present as number (%) or mean (standard deviation)

Tachycardia and bradycardia were defined as HR > 100/min and HR < 60/min, respectively

*SpO*_*2*_ Peripheral capillary oxygen saturation, *HR* Heart rate

### The relationship between remifentanil use and recovery time

In both univariable and multivariable analyses, the use of remifentanil was associated with more rapid recovery than was the use of midazolam/propofol (unadjusted odds ratio = 7.70, 95% confidence interval = 2.74–21.65; adjusted odds ratio = 15.88, 95% confidence interval = 4.14–60.90) (Table 4).

**Table 4:** Factors associated with rapid recovery in patients undergoing sedative bronchoscopy

	Univariable analysis		Multivariable analysis	
	Unadjusted OR (95% CI)	*p*	Adjusted OR (95% CI)	*p*
Age, yr	0.98 (0.95–1.01)	0.131	0.97 (0.93–1.001)	0.059
Female	0.71 (0.30–1.71)	0.448	0.53 (0.18–1.54)	0.241
Body mass index, kg/m^2^	0.93 (0.93–1.11)	0.772	0.92 (0.82–1.04)	0.195
COPD or asthma	0.37 (0.07–2.01)	0.249	0.93(0.11–7.76)	0.946
Chronic heart disease	2.74 (0.76–9.91)	0.123	0.62 (0.09–4.32)	0.628
Indication				
Others	Ref		Ref	
Lung cancer	0.53 (0.15–1.95)	0.343	0.69 (0.13–3.66)	0.665
Infection	0.90 (0.31–2.59)	0.841	1.43 (0.40–5.05)	0.581
Interstitial lung disease	0.98 (0.22–4.28)	0.976	1.72 (0.29–10.06)	0.548
Procedure				
Inspection only	Ref		Ref	
Inspection with bronchoscopic biopsy	0.92 (0.25–3.39)	0.896	0.27 (0.04–1.64)	0.154
Inspection with BAL and/or TBLB	0.92 (0.19–4.40)	0.913	0.67 (0.10–4.55)	0.684
Sedative drug				
Midazolam/propofol	Ref		Ref	
Remifentanyl	7.70 (2.74–21.65)	< 0.001	15.88 (4.14–60.90)	< 0.001

Data are present as a ratio (95% CI)

*OR* Odds ratio, *CI* Confidence interval, *COPD* Chronic obstructive pulmonary disease, *BAL* Bronchoalveolar lavage, *TBLB* Transbronchial lung biopsy, *Ref* Reference

## Discussion

We evaluated 186 subjects who underwent flexible bronchoscopy under sedation. Approximately 60% of these patients received remifentanil, while the remaining 40% received a combination of midazolam and propofol for procedural sedation. The patients in the remifentanil group were more likely to undergo invasive procedures during bronchoscopy (such as bronchoscopic biopsy, bronchoalveolar lavage, or transbronchial lung biopsy) than those in the midazolam/propofol group. Despite this finding, there was no significant difference between the groups in the occurrence of adverse safety events, including oxygen desaturation, hypotension, and arrhythmia. In addition, the recovery time was significantly shorter in the remifentanil group than in the midazolam/propofol group.

Ideal drugs for procedural sedation and analgesia (PSA), or “conscious sedation,” have a rapid onset and short duration of action, and maintain hemodynamic stability with no major side effects [14]. The classically used sedative drugs include the single or combined use of benzodiazepines, propofol, and opioids. A 2003 United Kingdom survey found that 78% of bronchoscopists routinely use midazolam sedation alone and that midazolam plus fentanyl/alfentanil was the most frequently combined regimen [15]. Another study found that the most commonly used drugs in Switzerland were midazolam (46%) and propofol (77%) [5]. However, unfortunately, there are no clear recommendations favoring one sedation regimen over another. The introduction of new sedative drugs, such as remifentanil and dexmedetomidine, has added to the complexity of combining sedative regimens [7–10].

Analgosedation refers to the use of an analgesic drug (usually an opioid) before a sedative is used to reach the sedative goal [16]. Analgosedation is preferred over the sedative-hypnotic approach in critically ill patients, for whom the primary goal is to address pain and discomfort before adding hypnotic agents [16]. The use of analgosedation has increased substantially over time and is accepted as a common protocol in the management of ICU patients in many hospitals [16]. Accumulating data have suggested that analgosedation is safe and effective for the management of very vulnerable ICU patients. Although these data also suggest that analgosedation is safe for use during fiberoptic bronchoscopy, there is little evidence supporting this practice.

Remifentanil is a potent, selective µ-opioid receptor agonist that is metabolized by blood and tissue esterases independent of organ function [17]. Remifentanil has a rapid onset and a short duration of action (half-life < 10 min) with no tissue accumulation. The advantage of this drug is that its effect does not last long after it is stopped. Thus, one of the most important advantages of this drug is that it can be safely used in patients with organ impairment such as acute kidney injury and severe liver disease [18–20]. It also provides good hemodynamic stability [21]. Another advantage of remifentanil is that it effectively suppresses cough [22], which seems suitable for PSA during fiberoptic bronchoscopy.

Interestingly, despite several advantages of remifentanil, its utility for PSA in patients undergoing fiberoptic bronchoscopy has not been well elucidated. Two studies showed that the use of remifentanil alone is safe in ICU patients [9, 10]. Another study showed that remifentanil attenuates the hemodynamic response to rigid bronchoscopy without an increase of hypotension or bradycardia [23]. However, most other studies regarding remifentanil studied its use in combination with other drugs [7, 24, 25]. For example, Ryu and colleagues showed that the use of remifentanil plus propofol was associated with a shorter recovery time than was that of dexmedetomidine. However, the combination of remifentanil and propofol during bronchoscopy led to a higher incidence of desaturation and a need for oral cavity suctioning than did dexmedetomidine use [7]. Therefore, it is unclear whether remifentanil can be safely and effectively used alone for PSA during fiberoptic bronchoscopy. From this perspective, our study provides important evidence that remifentanil can be used alone for PSA during fiberoptic bronchoscopy including cases that require complex procedures. In addition, we found that remifentanil use was associated with faster recovery time and comparable adverse events to those of midazolam/propofol use. The reasons for shorter procedure duration with remifentanil relative to midazolam/propofol are not fully explainable. Given the shorter half-life and stability of remifentanil compared to those of midazolam/propofol, the attending physicians might have given enough remifentanil to allow patients to be sufficiently sedated during bronchoscopy. On the other hand, patients receiving midazolam/propofol with relatively long half-lives may have been less sedated than those receiving remifentanil. However, since we did not compare the depth of sedation between the two groups, further studies are needed to confirm this suggestion.

One strength of our study is that it raises important questions regarding the clinical usefulness of remifentanil use alone for analgosedation during fiberoptic bronchoscopy. To the best of our knowledge, this is the first study to comprehensively evaluate the clinical utility and safety of remifentanil use in fiberoptic bronchoscopy in comparison with those of midazolam/propofol. Another strength of our study is that we employed a larger study population than did prior studies that evaluated the use of remifentanil alone during fiberoptic bronchoscopy. Regardless, this study also has several limitations. First, it has an inherent bias due to its retrospective design. It was also performed in a single university hospital. The second limitation is that the analgosedation drugs used in each patient were chosen at the discretion of the attending physicians. Therefore, this discretion may have resulted in the more frequent use of remifentanil (over conventional drugs) in vulnerable patients or in those who required more complex procedures. Therefore, the remifentanil group was more prone to include patients with disadvantageous clinical characteristics than was the midazolam/propofol group. Despite this limitation, we found that there were no significant differences between the groups with regard to adverse events. Third, we were unable to assess patient satisfaction scores and the level of sedation, which are important measures. Fourth, we could not assess the total dose of topical lidocaine used during the procedures.

## Conclusions

Despite a higher proportion of patients who underwent more invasive procedures in the remifentanil group than in the midazolam/propofol group, there was no significant difference in the occurrence of safety events between the groups. Furthermore, the recovery time was faster in the remifentanil group than it was in the midazolam/propofol group. Our findings suggest that remifentanil alone may be safely and effectively used for PSA during fiberoptic bronchoscopy. Further prospective comparative evaluation is required to establish its superiority and cost-effectiveness over other contemporary drugs.

## Data Availability

All data extracted in this study are included in this article.

## Abbreviations

CI: Confidence interval
ICU: Intensive care unit
IQR: Interquartile range
PSA: Procedural sedation and analgesia
SD: Standard deviation
